# Do Minority-Serving Institutions Make a Difference in the Engagement and Outcomes of Minority College Students? Empirical Evidence from China

**DOI:** 10.3390/bs15010096

**Published:** 2025-01-20

**Authors:** Yuheng Huang, Zengchen Han, Yu Tian, Yannan Cao

**Affiliations:** 1 College of Education, Capital Normal University, Beijing 100048, China; 2 Graduate School of Education, Beijing Foreign Studies University, Beijing 100089, China

**Keywords:** minority-serving institution, ethnic minority college students, learning psychology, learning behaviors, student engagement, propensity score matching

## Abstract

This study uses nationally representative data from the Chinese College Student Survey (CCSS) (*N* = 37,508) to examine the impact of minority-serving institutions (MSIs) on learning opportunities, processes, and outcomes for ethnic minority college students. The CCSS uses a self-report questionnaire with multiple scales to measure ethnic minority students’ development, including family and ethnic background, university admission opportunities, learning behavior and psychology, and skill development in areas such as leadership and innovative thinking. We employ logistic regression and propensity score matching and find that MSIs offer valuable learning opportunities to minority students from ethnic areas and economically disadvantaged backgrounds, as well as those with weak academic preparation. Furthermore, these institutions enhance ethnic minority students’ engagement in active and cooperative learning, participation in high-impact educational activities, acquisition of knowledge and skills, innovative thinking, leadership development, and overall growth. However, MSIs are less effective at fostering interpersonal relationships. Additionally, MSIs exert a significant positive influence on college students from ethnic groups characterized by strong religious affiliations or low average educational levels, particularly in terms of learning behaviors and both academic and social integration.

## 1. Introduction

Owing to ethnic minority students’ unique cultural and socioeconomic backgrounds, their academic success in higher education has long been a significant concern in the field of higher education research. As higher education has expanded globally, ethnic minority students have gained greater access to universities. This expansion has significantly advanced social equity and racial equality. The diverse religious beliefs, lifestyle habits, learning styles, and career aspirations that ethnic minority students bring to campuses have created richer multicultural learning environments. While this diversity has broadened universities’ intellectual and research horizons, it has also challenged traditional approaches to college education. Many countries have established higher education institutions that specifically cater to minority students, such as historically Black colleges and universities (HBCUs) for Black students, Hispanic-serving institutions for Latino students, and tribal colleges and universities for Native Americans in the United States. Mainland China also has minority-serving institutions (MSIs) that target minority college students for enrollment. Fifteen comprehensive MSIs and multiple higher education institutions have been established in ethnic regions with the aim of training minority students. From a policy standpoint, the government often provides preferential support for MSIs in terms of funding and projects. However, the literature reflects some debate regarding the impact of MSIs on the academic success of minority college students. While some researchers have argued that attending such institutions can lead to more successful learning outcomes and social success because they provide a learning environment closer to students’ family or community environments (Boland et al., 2021; Espinosa et al., 2017; Harmon, 2012; Kim & Conrad, 2006; Melguizo, 2008), others believe that attending ordinary universities may be more conducive to the integration of minority students into mainstream society (Guiffrida, 2003; Tinto, 2012). The impact of MSIs on the success of minority college students therefore remains unclear. To this end, this study utilizes survey data collected from the China College Student Survey (CCSS) to analyze the learning opportunities, processes, and outcomes of minority students in MSIs in mainland China. Ordinary universities are used as a control group to explore the significant value of MSIs in the development of minority college students.

## 2. Literature Review

The relationship between MSIs and the learning development of minority college students is an important research topic in the field of ethnic education. Since the 1960s, scholars in British and American educational anthropology have focused on the impact of school institutions on the learning and development of ethnic minority students and have proposed a variety of explanatory theories (Philips, 1992; Ausubel, 1964; McDermott, 1977; Spindler, 1987; Valentine, 1968). Scholars first focused on the differences between the cultures of ethnic minority families and schools. Theories such as cultural interruption, cultural deprivation, cultural conflict, and differences in language types emphasize the cultural differences between ethnic minority families and White-dominated schools in terms of language, learning styles, values, etc., arguing that these differences or conflicts lead to relatively low academic achievement among ethnic minority students. However, with the emergence of more research evidence, theories analyzing the impact of school institutions on the learning and development of ethnic minority students from a cultural perspective have faced criticism (Kuperminc et al., 1997; Rowley & Moore, 2002). In the field of higher education, Title III of the Higher Education Act of 1964 in the United States recognized a group of institutions dedicated to providing educational services to ethnic minorities (mainly Black and Native American students at that time), and research on the relationships between school institutions and the learning and development of ethnic minority college students entered a new stage of development. Since the 1980s, the research perspective has shifted from being dominated by anthropology to various theories from anthropology, sociology, psychology, and pedagogy. Furthermore, the research focus has shifted from ethnic culture to education itself. Research has analyzed the development of minority students in MSIs and non-MSIs, exploring the impact and unique value of different institutions on minority students’ learning and development. The research paradigm, which includes a variety of social science research methods, is based on qualitative material inductive theory or survey data to construct an empirical model. Research findings from the past two to three decades can be divided into two main areas examining how different types of institutions affect minority college students’ learning and development. The first area analyzes how institutional factors in general education settings—including language, racial discrimination, campus climate, and institutional support—influence ethnic minority students’ graduation rates and academic achievement. The second area examines whether specialized institutions such as MSIs can enhance ethnic minority students’ learning and development.

### 2.1. The Relationship Between Institutions and the Educational Success of Minority Students

Proponents assert that specific elements of school institutions, especially school culture and campus discrimination, exert either positive or negative impacts on minority students. Sherman et al. (1994) reported that when the ethnic living environment of minority students prior to college is similar to their college environment, these students can more effectively integrate academically and socially, exhibiting a greater level of academic adaptability. Solorzano et al. (1999) interviewed students from three historically Black universities and discovered that campus prejudice and discrimination resulted in negative academic behaviors among minority students, such as dropping courses, changing majors, and even transferring to other universities. Hurtado and Ponjuan (2005) reported that in a university setting dominated by European cultural traditions, cultural conflicts are likely to affect the learning psychology of Latino students and lead them to doubt their ability to succeed in college. Wei et al. (2011) reported that the institutional environment serves as an intermediary variable between academic stress and learning attitudes among minority students; thus, creating a multicultural and welcoming campus environment helps increase minority students’ academic persistence. Whether they attend predominantly White or Black colleges, African American college students need to integrate actively into the campus environment to adapt better to college life (Adan & Felner, 1995).

Teachers are among the most important factors influencing the growth of ethnic minority students. Atkins et al. (2014) reported that increasing the representation of African American and Latino/a teachers increases educational expectations for African American students, whereas greater Latino/a teacher representation improves both school connectedness and educational expectations for Latino/a students. Research has also demonstrated the importance of racial matching between teachers and students for academic achievement. Dee (2004) reported that students taught by teachers of their own ethnicity tend to achieve better grades in mathematics and reading. Similarly, Egalite et al. (2015) reported small but significant positive effects when Black and White students were assigned to same-race teachers in reading, and when Black, White, and Asian/Pacific Islander students had same-race/-ethnicity teachers in math—with these benefits being particularly pronounced among lower-performing Black and White students. Additional studies have shown that Black teachers are more effective instructors for Black students (Hanushek et al., 2004) and that teacher race significantly impacts student performance on standardized tests (Oates, 2003). These findings suggest that creating a multicultural educational environment through minority teacher representation contributes to minority student success (Hue & Kennedy, 2014).

Findings from research on K-12 education also affirm the importance of the school environment. Thomas and Collier (2002) conducted a study that tracked minority students in both a bilingual school and a monolingual school and reported that after four to seven years of bilingual education, minority students demonstrated academic performance superior to that of their peers in the monolingual school across all subjects. The authors suggested that offering a school environment that provides sociocultural support to minority students is crucial and contributes to the development of native language, academic, and cognitive skills among students who use minority languages. In another study, Gardner-Kitt (2005) investigated the learning and development of 140 Black middle school students and concluded that the school environment serves as a mediating factor between ethnic identity and academic performance. The authors suggested that schools can affect minority students’ learning behavior and, consequently, their academic achievement by encouraging them to develop national self-confidence and respect for different cultures.

However, contradictory findings indicate that ethnic minority and nonminority students are equally impacted by the school environment, although these studies are less numerous than those supporting the opposite view. These findings suggest that ethnic minority students do not experience a distinct “specificity” of school influence and that their academic performance is attributed primarily to their personal characteristics rather than the school environment. In an analysis of the 1988 National Educational Longitudinal Study (NELS) and its 1990 follow-up data, Ehrenberg et al. (1995) reported that teacher characteristics, including race, do not affect student test scores. In their research on the relationship between campus bias and college students’ learning adaptability, Cabrera et al. (1999) reported that discriminatory behaviors have detrimental effects on minority students’ academic and intellectual development, social experiences, and intentions. However, the predictive factors for academic achievement among both minority and nonminority students are essentially the same. In terms of cognitive performance and academic persistence, minority students are influenced primarily by their individual academic abilities and do not show a significant correlation with the school environment. On the basis of an analysis of 24 quantitative studies, Driessen (2015) found little unambiguous empirical evidence that stronger ethnic matching—whether through one-to-one teacher-student ethnic pairing or a larger proportion of ethnic minority teachers at an ethnically mixed school—leads to predominantly positive results.

### 2.2. The Relationship Between MSIs and the Learning Achievement of Minority College Students

A substantial body of supportive evidence indicates that MSIs have a positive effect on the academic development of minority students. In a landmark study, Upton and Fleming (1984) reported that HBCUs were more effective than predominantly White institutions (PWIs) in developing the skills Black students need to adapt to postuniversity society, such as self-confidence, motivation, high aspirations, and the ability to thrive in competition. Since then, other studies have reached similar conclusions, demonstrating that HBCUs offer a more supportive and positive learning environment for African American students than PWIs do (DeSousa & Kuh, 1996; Flowers & Pascarella, 1999; Watson & Kuh, 1996). Using a quasiexperimental research approach, Terenzini et al. (1997) measured the cognitive levels of two groups of students entering traditional Black colleges and predominantly White colleges. They reported that despite significant differences in the backgrounds of the two groups in terms of their precollege personal characteristics and learning experiences, students entering traditional Black colleges spent more time studying, preferred to live on campus, and received more academic support from their peers. Research on Chinese ethnic colleges provides additional supporting evidence. Luo et al. (2021) reported that ethnic minority students studying in MSIs have a significantly greater sense of learning achievement than Han students, China’s main ethnic group. Similarly, another study revealed that ethnic minority students in MSIs experienced significantly greater levels of academic challenge than Han students; however, among minority groups, Hui and Manchu students reported significantly lower perceptions of academic challenges than other ethnic groups (Wang & Zou, 2016).

According to a report from the American Indian Higher Education Association, the experience at tribal colleges and universities has a significant positive effect on the lives of American Indian students within the wider American Indian community (Merisotis & McCarthy, 2005). Brown et al. (2003) reported that American Indian students who transferred from a two-year tribal college to a four-year university were very satisfied with their experience at the tribal college, suggesting that beginning at a tribal college and subsequently transferring to a four-year university is an effective method for academic advancement among American Indian students. Similarly, Fosnacht and Nailos (2015) revealed that Hispanic institutions had a favorable impact on the academic engagement and self-cognitive ability of Hispanic students.

Several studies have challenged the positive impact of MSIs on minority students. Kim and Conrad (2006) reported no noteworthy difference in the likelihood of Black students obtaining a bachelor’s degree, regardless of whether they attended traditional Black colleges (HBCUs) or predominantly White colleges (PWIs). Nevertheless, a greater percentage of Black college students engaged in teacher research, indicating that traditional Black colleges may provide more academic opportunities for Black students. Moreover, Nelson Laird et al. (2007) reported that, compared with their counterparts at predominantly White colleges, Hispanic college students at a Hispanic college showed no substantial disparities in learning engagement, satisfaction, or self-reported educational gains among lower grades. Park and Flores (2013) used regression modeling and reported that minority identity is a significant predictor of MSI enrollment opportunities after controlling for certain background features. However, after controlling for school characteristics, the effect of ethnicity on graduation rates disappeared, and there were no notable differences in graduation outcomes between Hispanic and Black students who attended four-year colleges and their White counterparts (Flores & Park, 2015). Flores et al. (2017) utilized propensity score matching to analyze the graduation rates of African American and Latino students in MSIs and non-MSIs and concluded that while ethnic colleges offered admission opportunities to minority students with weaker educational backgrounds, after matching students with similar backgrounds, MSIs had no sustained positive or negative impact on the graduation outcomes of minority students.

Research has focused predominantly on two primary issues: the sensitivity of minority students to school education and the overall impact of MSIs on the learning and development of minority college students, including the mechanisms underlying this influence. Studies on the first issue have generally reached more consistent conclusions concerning the positive impact of a supportive atmosphere toward ethnic culture within educational institutions on minority students and, conversely, the detrimental effects of an environment characterized by ethnic discrimination and prejudice. Obviously, the response to the second question is more complicated. Renowned American educationalists Mayhew et al. (2016) analyzed this complexity in their master work, “How Universities Affect Students”. They highlighted that MSIs exert distinct impacts on the learning and development of minority college students across various domains. Currently, MSIs contribute to improvements in graduation rates, self-awareness, and other psychological indicators among ethnic minority students. However, their effects on learning, cognition, leadership, work life, and income remain less clear. This second aspect involves the influence mechanisms of MSIs, suggesting that such institutions may offer a more supportive environment with teachers and peers compared to predominantly White colleges. Overall, attending minority or racially diverse schools, such as Black or Latino colleges, produces results for minority students that are at least as favorable as those from other schools, which usually range from positive to negligible effects.

## 3. Analytical Framework and Research Hypotheses

In traditional research, the success of ethnic minority college students has been primarily defined by quantifiable student attainment indicators, such as enrollment in postsecondary education, grades, persistence to the sophomore year, length of time to degree, and graduation (Venezia et al., 2005). In recent years, scholars have broadened the definition of student success within college student development theory, recognizing that success extends beyond visible learning achievements to include implicit learning psychology, learning behavior, and emotional investment. Studies have shown that student success also encompasses satisfaction with one’s experience, as well as feeling comfortable and validated in the learning environment (Hossler & Vesper, 1999; Strauss & Volkwein, 2002). Minority students’ success is further linked to numerous desired student and personal development outcomes that benefit both individuals and society. These outcomes include proficiency in writing, speaking, critical thinking, scientific literacy, and quantitative skills, as well as advanced levels of personal development, such as self-awareness, confidence, self-worth, social competence, and a sense of purpose (Kuh et al., 2006; Pascarella & Terenzini, 2005).

To better analyze how MSIs affect ethnic minority students’ success, we draw inspiration from the theories of Kuh and other scholars to examine success through three lenses: learning opportunities, learning processes, and learning outcomes. Learning opportunities represent students’ ability to gain university admission. The learning process focuses on student learning engagement, which refers to their behaviors and perceptions regarding participation in various education-related activities both inside and outside the classroom. This study analyzes learning engagement through two dimensions, learning psychology and learning behavior, which are measured by learning motivation, interpersonal relationships, active and cooperative learning, and high-impact educational activities. Learning outcomes concentrate on learning attainment—students’ improvement in knowledge, skills, cognitive thinking, emotional values, and other aspects after their university education. Learning attainment is divided into academic development and social integration and is measured through knowledge and skills, innovative thinking, organizational leadership, and learning satisfaction.

Prominent theories in the literature have established that ethnic background, family background, and educational preparation play pivotal roles in shaping the learning and development of ethnic minority college students. Ethnic and family backgrounds, which have the most lasting effect on individual growth, are inherent factors that act as exogenous variables in students’ growth. These factors shape students’ learning attitudes and values through the educational expectations and parenting styles of ethnic groups and affect their educational attainment through language, economic conditions, and other long-lasting influences. The education level, religious beliefs, ethnic language, and economic and social status of ethnic groups impact the educational readiness of ethnic minority students prior to university enrollment, as well as students’ selection of MSIs, which subsequently influences their learning inputs and gains during their university education.

Both precollege experience and MSIs are considered inherent attributes that are subject to change through postnatal efforts after the removal of early inherent factors. The precollege experience encompasses the educational preparation of college students before enrollment. It primarily shapes students’ learning abilities, habits, and styles, impacting their access to learning opportunities at universities, as well as their learning processes and outcomes during their academic journey. MSIs influence the learning and development of ethnic minority college students by lowering admission standards and offering culturally diverse courses.

**Hypothesis 1 (H1).**  *Significant disparities in residential areas, family economic and social status, and Gaokao (National College Entrance Examination) scores are expected between ethnic minority college students enrolled in MSIs and those enrolled in non-MSI programs.*

**Hypothesis 2 (H2).**  *The differences between MSIs and non-MSIs in the creation of a multicultural campus environment and the employment of minority teachers lead to notable distinctions in the learning engagement and attainment of minority college students across the two types of institutions.*

**Hypothesis 3 (H3).**  *The impact of MSIs on the college success of ethnic minority students varies among different ethnic groups due to differences in education, language, and religion across the 55 ethnic groups in China.*

## 4. Research Design

### 4.1. Data Sources

This study uses nationally representative data from the 2011–2016 dataset of the China College Student Survey (CCSS), which is adapted from the National Survey of Student Engagement (NSSE). The NSSE was originally developed in the United States and later indigenized for the Chinese higher education context. Annually, the CCSS employs stratified random sampling within participating institutions, taking into account grade, discipline, and gender. The sample for this study comprised 16 universities from Project 985 and 27 from Project 211 (both projects are government-funded initiatives aimed at developing world-class universities; universities selected for these projects are known for their superior research and teaching quality in China), as well as 68 general undergraduate institutions, including 15 MSIs. The sample represented all 55 ethnic minority groups in China.

This study examines the influence of MSIs on college students from diverse ethnic groups by analyzing the collective impact of these institutions on ethnic minorities and selecting specific groups from among the 55 ethnic minority groups in China. The selection of these ethnic groups is based on three variables: average ethnic educational level, language, and religion. This study identifies Korean, Mongol, Hui, Zhuang, Uyghur, Miao, Yi, and Zang, listed in descending order of average years of education, as belonging to different language and religious influence groups, as depicted in Table 1.

### 4.2. Basic Information on the Measurement Model and Variables

The primary econometric models employed in this study are logistic regression (logit) and propensity score matching (PSM). The logit model is used to analyze the background characteristics of ethnic minority college students who enroll in MSIs and non-MSIs. This analysis aims to derive the distribution characteristics of admission opportunities to MSIs among students from diverse backgrounds to address the question of “who enters MSIs”.

(1) $$logit(p)=Ln\left(\frac{p}{1-p}\right)=\alpha +\sum_{i=1}^{I}\beta_{i,n}X_{i,n}+\sum_{j=1}^{J}\beta_{j,n}X_{j,n}+\sum_{z=1}^{Z}\beta_{z,n}X_{z,n}+\varepsilon$$

In Equation (1), *p* denotes the likelihood of enrolling in a minority-serving institution, whereas *p*/(1 − *p*) indicates the ratio of the likelihood of enrolling in an MSI to the likelihood of enrolling in a non-MSI. Moreover, *n* denotes a specific ethnic group. The explanatory variables *X_i_*, *X_j_*, and *X_z_* correspond to precollege experience, family background, and control variables, respectively. The regression coefficients of the corresponding explanatory variables are represented by *β_i_*, *β_j_*, and *β_z_*. A positive coefficient signifies that the explanatory variable promotes the enrollment of college students in MSIs, with a larger coefficient indicating a greater likelihood of enrollment in these institutions. α denotes the intercept term of the equation.

The PSM model is used to assess the impact of MSIs on the learning engagement and attainment of ethnic minority college students. Chinese higher education institutions employ selective enrollment methods that are primarily based on high school students’ *Gaokao* scores. However, MSIs offer support for ethnic minority students by prioritizing their admission based on the same examination scores. Therefore, there may be significant differences between the backgrounds of students at MSIs and non-MSIs, which could lead to a potential endogeneity problem when a regression model is used directly. Therefore, it is impossible to accurately judge the influence of MSIs on the success of minority college students. PSM is an important method used by researchers to solve the problem of endogeneity in variable relationships. PSM first calculates the propensity score of students who enroll in MSIs using a logistic regression model. It then uses matching techniques to identify corresponding individuals for each student entering both MSIs and non-MSIs. This process results in two types of student samples that are similar to the experimental group (MSIs) and the control group (non-MSIs). By employing the t test method or multivariate statistical analysis with the matched sample, an estimate of the average treatment effect can be obtained (As a result of the inaccuracy of the standard error derived from the PSM method, the bootstrap method was employed to conduct 50 sampling tests after the PSM model analysis. This approach was taken to accurately assess significance and standard error).

This study employed the CCSS survey questionnaire items to create analysis variables and converted the options into percentages for statistical analysis. Details of the analysis variables are presented in Table 2.

## 5. Research Findings

### 5.1. MSIs Provide Greater Opportunities for College Admission for Relatively Disadvantaged Minority Students

Table 3 shows the results of logistic regression analyzing the backgrounds of students in MSIs. The model is significant for ethnic minorities as a whole, as well as for each of the case ethnic groups. The results show that minority college students in MSIs and non-MSIs exhibit significantly different characteristics.

Overall, minority students from MSIs are more likely to come from ethnic enclaves, be female, study arts subjects, have parents with lower levels of education, and have lower *Gaokao* scores than their counterparts at non-MSIs. These significant variables have a similar effect on most of the ethnic groups studied. The variable of ethnic enclaves has a positive correlation, whereas parents’ occupational level and education level, as well as the student’s *Gaokao* scores, are negatively correlated. Compared with non-MSIs, MSIs provide more admission opportunities for minority college students from ethnic enclaves, as well as those with lower family economic and social statuses and lower *Gaokao* scores. Conversely, when students come from families with relatively poor economic conditions and insufficient educational preparation, MSIs may face greater difficulties in cultivating talent than non-MSIs do.

### 5.2. MSIs Promote Behavioral Learning Engagement Among Minority College Students

As presented in the logistic regression model in Table 3, there are differences in family and educational backgrounds between minority college students at MSIs and those at non-MSIs. To avoid bias in the regression results, kernel matching, which is a method of propensity score matching, is used to pair and compare minority college students from the two types of institutions on the basis of the results of the logistic regression to obtain the net effect of MSIs on minority college students’ learning input and gains.

Table 4 shows the average treatment effects of MSIs on the learning engagement of college students from different ethnicities. Compared with their counterparts in non-MSIs, ethnic minority students at MSIs exhibit the following characteristics: Hui students demonstrate stronger learning motivation; ethnic minority students overall, as well as Hui and Zhuang students, show weaker interpersonal relationships; ethnic minority students overall, as well as Uyghur and Zang students, display stronger proactive and cooperative learning behaviors in their coursework; and ethnic minority students overall, as well as Mongolian, Hui, Zhuang, Miao, and Zang students, participate in more high-impact educational activities outside the classroom. The results of the model show that MSIs have a relatively positive influence on the learning behaviors of ethnic minority college students, particularly regarding high-impact educational activities, but have a relatively weak influence on the psychological aspects of learning.

### 5.3. MSIs Enhance the Learning Attainment of Minority College Students

Table 5 shows the average treatment effects of MSIs on the learning attainment of college students from different ethnicities. Compared with their non-MSI counterparts, ethnic minority college students who attend MSIs exhibit the following characteristics: ethnic minority students as a whole, as well as Mongolian, Hui, Uyghur, and Zang students, achieve higher levels of knowledge and skills; ethnic minority college students as a whole achieve higher levels of innovative thinking; ethnic minority students as a whole, as well as Hui and Uyghur students, demonstrate stronger organizational leadership; and ethnic minority students as a whole, as well as Hui, Miao, and Yi students report higher levels of learning satisfaction. MSIs have a more positive effect on the learning attainment of ethnic minority college students than non-MSIs do, especially in terms of improvements in knowledge and skills.

## 6. Discussion and Conclusions

This study utilized data from the Chinese College Student Survey (CCSS) and a propensity score matching model to explore the impact of ethnic colleges on the learning opportunities, processes, and outcomes of ethnic minority college students. From the empirical results, it can be inferred that all three hypotheses proposed in this study have been confirmed, leading to the following conclusions.

First, MSIs provide university access for minority students from ethnic enclaves, students with relatively low family socioeconomic backgrounds, and students with weaker preparation in high school education through preferential admission methods such as score reductions. However, this means that MSIs face more difficulties in talent development and require more resources.

Second, MSIs have a valuable impact on the learning and development of minority college students. As shown in Table 6, MSIs have a more positive impact than non-MSIs on minority college students’ proactive and cooperative learning behaviors, participation in high-impact educational activities, learning satisfaction, improvement in knowledge and skills, innovative thinking, and organizational leadership. However, MSIs are less effective at promoting interpersonal relationships. With respect to the ethnicities considered, except for the Korean ethnicity, MSIs have a significant effect on the other seven ethnicities, particularly in terms of in-class and out-of-class learning engagement, academic development, and social integration, cultivating these skills more effectively than non-MSIs.

The findings of this study support the conclusion that MSIs positively influence the educational development of ethnic minority college students. This finding aligns closely with previous research by Upton and Fleming (1984), Flowers and Pascarella (1999), Terenzini et al. (1997), Kuh (2008), and Pascarella (2019). MSIs foster a campus culture that resonates with the familial and community environments of ethnic minority students by promoting a nondiscriminatory atmosphere and employing a greater number of ethnic minority faculty members. As a result, ethnic minority college students at MSIs exhibit greater engagement in active learning and achieve enhanced academic outcomes. However, a notable concern is that these students perceive a lower quality of interpersonal relationships. According to the theory of racial boundaries, the collision of different ethnic minority cultures may lead to more pronounced differences between racial groups within MSIs, which may hinder interaction and communication among college students of different races. Given that MSIs in mainland China are not exclusive to any particular ethnic group but admit students from diverse backgrounds, including the Han ethnicity, the complexity of this diversity may intensify cultural collisions and potentially hinder the formation of close relationships among students from different ethnic backgrounds. Nevertheless, whether assessed from the perspective of all non-Han ethnic groups in China or the eight selected case studies, the positive effects of MSIs on ethnic minority college students significantly outweigh the negative impacts.

This study supports Yosso’s Community Cultural Wealth Model, which views community cultural wealth as a collection of knowledge, skills, abilities, and contacts that communities of color use to survive and resist both macro and micro forms of oppression. Communities of color possess various forms of cultural wealth capital before formal education begins, including aspirational, social, navigational, linguistic, resistant, and familial capital (Yosso & Burciaga, 2016; Yosso, 2005). When schools effectively tap into the rich cultural wealth of ethnic minority communities, they can better support these students’ educational success. MSIs, by recognizing and building upon their students’ community cultural capital, provide more targeted support for ethnic minority students’ development. This approach helps explain why ethnic minority students often experience greater success in MSIs.

This study also supports Ogbu’s cultural model theory, which posits that both majority and minority ethnic groups use their distinct cultural values to interpret the content, context, and learning behaviors of group members during educational activities (Ogbu, 1982, 1987, 1991). Cultural differences and similarities influence the learning behaviors of students from various ethnic backgrounds, leading to varying academic outcomes. In mainland China, there are 55 recognized ethnic minority groups, each exhibiting different levels of interaction and integration with mainstream society. Additionally, these groups demonstrate differences in religious beliefs, educational attainment, and acceptance of the mainstream educational system. The impact of MSIs on ethnic minority college students varies accordingly. This study revealed that MSIs have a significantly more positive effect on ethnic groups with stronger religious beliefs or lower average education levels, whereas the effect is less pronounced for groups with weaker religious beliefs or higher average education levels.

This study has several limitations that future research could address. First, our findings show that MSIs are less effective than non-MSIs in fostering interpersonal relationships among ethnic minority college students. Specifically, Hui and Zhuang college students in MSIs reported significantly poorer interpersonal relationships, whereas other ethnic minority students in MSIs also reported relatively poor interpersonal relationships, although the difference was not statistically significant. Future research should examine this phenomenon more closely by investigating the factors influencing interpersonal relationships among ethnic minority students and how MSIs contribute to both positive and negative relationship outcomes. Second, while our data demonstrate MSIs’ effects on minority students’ learning success, they do not explain the mechanisms underlying these outcomes. For example, our analysis shows that religious minority college students experience higher levels of learning engagement in MSIs, but further research is needed to clarify the role of religious belief in their academic success. Finally, although we used the PSM method to establish causal relationships, additional undiscovered variables may significantly impact minority students’ learning experiences in MSIs. Future research should employ more comprehensive data analysis and qualitative methods to better understand how MSIs influence the learning behaviors and psychology of ethnic minority college students.

## Figures and Tables

**Table 1:** Basic sample information.

Ethnic Characteristics		Ethnic Minorities	Korean	Mongol	Hui	Zhuang	Uyghur	Miao	Yi	Zang
Main distribution areas		Mainland China	Northeast China	Northeast/Northwestern China	Northwestern China	South China	Northwestern China	South China	Southwestern China	Southwestern China
Primary language		N/a	Chinese/minority language	Chinese/minority language	Chinese	Chinese/minority language	Minority language	Chinese	Chinese/minority language	Minority language
Religion		N/a	N/a	Buddhism	Islam	N/a	Islam	N/a	N/a	Buddhism
Average years of education		N/a	10.25	9.17	8.14	8.12	8.00	7.17	6.49	5.33
University type	Non-MSI	83.86	64.19	84.89	78.27	90.11	81.24	85.63	75.15	63.62
	MSI	16.14	35.81	15.11	21.73	9.89	18.76	14.37	24.85	36.38
Gender	Female	52.06	71.86	56.95	51.95	53.67	58.9	44.3	50.82	59.91
	Male	47.94	28.14	43.05	48.05	46.33	41.1	55.7	49.18	40.09
Concentrated area of nationalities	No	57.60	74.53	70.17	76.95	28.09	39.12	40.9	41.77	36.46
	Yes	42.40	25.46	29.82	23.04	71.9	60.88	59.1	58.23	63.54
Sample		37,508	726	3891	4946	4740	1311	2018	978	1556

**Table 2:** Variable descriptions.

Variable Type	Variable Name	Cronbach’s Alpha	Question Items and Assignments
Learning Engagement: Psychology of Learning	Learning Motivation	0.70	(Description of learning, 1—strongly agree, 4—strongly disagree): I will find ways to overcome difficulties in learning; I am filled with joy when I concentrate on learning; I am willing to learn because it makes me grow; sense of meaning in learning. Overall level of motivation to learn (1—very weak, 7—very strong). Level of interest in the profession (1—very interested, 4—not interested at all). Professionalism promotes a satisfying life (1—very helpful, 4—not helpful at all).
	Interpersonal Relationships	0.81	(Interpersonal descriptions, 1—very good, 7—very poor): classmates; class instructors; student system staff such as counselors; administrators such as the registrar’s office.
Learning Engagement: Learning Behaviors	Active and Cooperative Learning	0.83	(Frequency of behavior, 1—very often, 4—never): Take the initiative to ask questions or participate in discussions in class; thinking and responding positively in class to questions for which there is no set answer; giving a presentation on a research topic in class after thorough preparation; collaborating with other classmates in completing a course task or homework assignment; helping other classmates to understand the content of the course; questioning the instructor’s viewpoints in class; taking focused notes in class; listening attentively to the instructor in class; discussing course content with classmates after class.
	High-Impact Educational Activities	0.66	(Activities already engaged in, 1—already done, 4—undecided): internships, social practices or surveys; community service or volunteering; submitting articles to professional and academic journals; participating in study clubs (e.g., book clubs, English clubs); doing research with class instructors; participating in academic, professional, entrepreneurial, or design competitions; enrolling in professional credentials/skills grade certificates.
Learning Attainment: Academic Development	Knowledge and Skills	0.86	(University enhancement, 1—very strong, 7—very weak): Having a wide range of knowledge areas; specialized knowledge and skills; oral expression; written expression; ability to use information technology; ability to analyze data and statistical information.
	Innovative Thinking	0.84	(College enhancement, 1—very strong, 7—very weak): Critical thinking; solving complex problems in reality; applying innovative ideas or approaches to problem solving; flexibility.
Learning Attainment: Social Integration	Organizational Leadership	0.68	(College enhancement, 1—very strong, 7—very weak): Organizational leadership; ability to work effectively with others.
	Learning Satisfaction	0.81	(Satisfaction, 1—very satisfied, 7—very dissatisfied): Overall college attendance experience; overall personal gains and growth while in school.
Family Background	Ethnic Enclaves	N/a	Whether the family’s area is located in an ethnic enclave: inhabited area—1; noninhabited area—0
	Parental Occupation	N/a	Categorized into five groups: unemployed, peasantry, blue-collar workers, white-collar workers, and gold-collar workers.
	Parents’ Years of Education	N/a	0–20 years, from no schooling to graduate school.
	Annual Family Income	N/a	Logarithm of annual family income.
Educational Preparation	Gaokao Score	N/a	Gaokao scores are converted to standardized scores based on province, year, and category of candidate.
	Liberal Arts	N/a	Subjects for Gaokao: liberal arts—1; science—0
Types of Institution	MSIs	N/a	Students enrolled in MSIs or non-MSIs: MSIs—1; non-MSIs—0
Control Variable	Universities Included in Project 985	N/a	Institution type: Institution belongs to the “985 Project Construction University”—1; otherwise—0
	Gender	N/a	Student self-reported gender: male—1; female—0

**Table 3:** Logistic regression modeling of MSI student backgrounds.

	(1)	(2)	(3)	(4)	(5)	(6)	(7)	(8)	(9)
	Ethnic Minorities	Korean	Mongol	Hui	Zhuang	Uyghur	Miao	Yi	Zang
Ethnic enclaves	0.166 ***	0.267	0.120	0.159	0.0110	0.0749	0.646 ***	0.115	−0.212
	(0.0378)	(0.292)	(0.147)	(0.101)	(0.130)	(0.285)	(0.151)	(0.203)	(0.164)
Peasantry	0.125	−0.677	−0.171	0.356	0.279	0.149	−0.0243	0.272	0.725 **
	(0.0794)	(0.682)	(0.288)	(0.205)	(0.320)	(0.493)	(0.303)	(0.413)	(0.274)
Blue-collar workers	0.126	−0.995 *	−0.231	0.128	0.226	0.190	0.271	0.225	0.183
	(0.0770)	(0.446)	(0.278)	(0.191)	(0.319)	(0.520)	(0.285)	(0.427)	(0.289)
White-collar workers	0.0540	−0.615	−0.247	−0.0964	0.488	−0.169	0.292	0.292	0.663
	(0.0877)	(0.530)	(0.297)	(0.220)	(0.352)	(0.564)	(0.336)	(0.482)	(0.350)
Gold-collar workers	0.0862	−1.263 *	−0.405	0.0381	0.0527	−0.391	0.522	0.625	0.000781
	(0.0857)	(0.509)	(0.289)	(0.213)	(0.355)	(0.602)	(0.334)	(0.465)	(0.326)
Parental years of education	−0.0222 ***	−0.117 *	0.0197	−0.0306 *	−0.00297	0.0435	−0.0653 **	−0.0184	−0.0146
	(0.00575)	(0.0464)	(0.0223)	(0.0132)	(0.0220)	(0.0365)	(0.0240)	(0.0322)	(0.0180)
Family income	−0.00671	0.0499	−0.0341	−0.0211	0.0651	0.0110	0.0283	−0.0620	0.0338
	(0.0114)	(0.0769)	(0.0339)	(0.0260)	(0.0476)	(0.0879)	(0.0546)	(0.0688)	(0.0465)
Gaokao score	−0.340 ***	−1.063 ***	−0.571 ***	−0.380 ***	0.0119	−0.157	−0.415 ***	−0.444 ***	−0.194 **
	(0.0190)	(0.179)	(0.0675)	(0.0485)	(0.0685)	(0.147)	(0.0774)	(0.109)	(0.0664)
Liberal arts	1.243 ***	1.709 ***	1.385 ***	0.898 ***	1.223 ***	3.583 ***	0.946 ***	1.087 ***	1.491 ***
	(0.0369)	(0.304)	(0.134)	(0.0854)	(0.120)	(0.365)	(0.146)	(0.194)	(0.154)
Universities included in Project 985	2.937 ***	5.426 ***	3.991 ***	2.521 ***	2.524 ***	4.265 ***	2.707 ***	3.431 ***	3.169 ***
	(0.0391)	(0.418)	(0.156)	(0.0935)	(0.129)	(0.315)	(0.166)	(0.225)	(0.187)
Male	−0.530 ***	−0.531	−0.750 ***	−0.454 ***	−0.570 ***	−0.308	−0.526 ***	−0.379 *	0.0236
	(0.0368)	(0.313)	(0.135)	(0.0860)	(0.124)	(0.267)	(0.145)	(0.187)	(0.145)
Intercept	−3.195 ***	−3.135 **	−3.870 ***	−2.169 ***	−4.133 ***	−7.174 ***	−3.287 ***	−2.737 ***	−4.173 ***
	(0.145)	(1.075)	(0.489)	(0.338)	(0.582)	(1.049)	(0.637)	(0.800)	(0.559)
Observed value	36,094	846	3665	4761	4569	1224	2465	1139	1476

Note: Standard errors in parentheses, *** *p* < 0.001, ** *p* < 0.01, * *p* < 0.05.

**Table 4:** Impact of MSIs on minority college students’ learning engagement (PSM model).

		(1)	(2)	(3)	(4)	(5)	(6)	(7)	(8)	(9)
		Ethnic Minorities	Korean	Mongol	Hui	Zhuang	Uyghur	Miao	Yi	Zang
Learning Motivation	MSI	64.415	58.297	64.615	64.963	63.451	69.576	64.059	64.076	64.219
	Non-MSI	64.272	61.841	64.478	63.672	63.768	68.009	63.556	63.505	63.323
	Difference in value	0.147	−3.544	0.146	1.406	−0.301	1.564	0.518	0.573	0.894
	bs standard error	0.314	3.058	1.053	0.758	0.981	2.105	1.211	1.826	1.025
	Z value	0.47	−1.16	0.14	1.85 *	−0.31	0.74	0.43	0.31	0.87
Interpersonal Relationships	MSI	65.024	65.083	66.323	65.308	61.175	68.907	62.592	65.647	64.712
	Non-MSI	66.547	63.946	66.991	66.964	65.043	65.184	64.710	64.921	64.187
	Difference in value	−1.523	1.137	−0.631	−1.560	−3.868	3.191	−2.178	0.725	0.437
	bs standard error	0.403	3.327	1.629	0.750	1.049	3.331	1.533	2.168	1.799
	Z value	−3.78 ***	0.340	−0.390	−2.08 **	−3.69 ***	0.960	−1.420	0.330	0.240
Active and Cooperative Learning	MSI	51.816	50.632	53.678	51.171	50.517	59.016	49.897	51.834	50.970
	Non-MSI	50.371	47.576	51.486	50.925	50.398	49.628	49.858	50.821	45.990
	Difference in value	1.446	3.059	2.151	0.336	0.157	9.512	0.091	1.014	5.025
	bs standard error	0.322	2.546	1.728	0.896	1.036	2.361	1.202	1.577	1.005
	Z value	4.50 ***	1.2	1.24	0.38	0.15	4.03 ***	0.08	0.64	5.00 ***
High-Impact Educational Activities	MSI	34.038	28.960	33.346	38.122	34.922	25.081	35.742	36.783	29.069
	Non-MSI	28.621	27.730	29.878	28.993	29.720	23.634	27.911	32.397	21.234
	Difference in value	5.416	1.229	3.516	9.166	5.277	0.932	7.908	4.391	7.514
	bs standard error	0.507	5.444	2.082	0.997	1.286	3.210	2.028	2.791	1.939
	Z value	10.67 ***	0.23	1.69 *	9.19 ***	4.1 ***	0.29	3.9 ***	1.57	3.88 ***

Note: *** *p* < 0.001, ** *p* < 0.01, * *p* < 0.05. The experimental group of the PSM model consists of all ethnic minority students in MSIs or ethnic minority college students in the case, whereas the control group includes the same group of people who attend non-MSIs. The matching variables for the logit model are ethnic enclave, parental occupational level, parental years of education, annual family income, and the standard *Gaokao* score.

**Table 5:** Impact of MSIs on learning attainment for minority college students (PSM model).

		(1)	(2)	(3)	(4)	(5)	(6)	(7)	(8)	(9)
		Ethnic Minorities	Korean	Mongol	Hui	Zhuang	Uyghur	Miao	Yi	Zang
Knowledge and Skills	MSI	58.027	53.907	59.912	58.287	55.611	65.291	55.465	58.018	56.696
	Non-MSI	56.425	57.073	57.455	56.261	55.159	60.762	54.927	57.765	51.757
	Difference in value	1.602	−3.188	2.466	2.047	0.491	4.593	0.544	0.256	4.816
	bs standard error	0.405	3.660	1.333	0.873	1.243	2.786	1.298	2.327	1.382
	Z value	3.95 ***	−0.87	1.85 *	2.34 **	0.39	1.65 *	0.42	0.11	3.48 ***
Innovative Thinking	MSI	59.803	57.369	61.846	60.882	57.935	65.922	57.913	59.228	56.480
	Non-MSI	58.771	52.959	61.568	59.775	57.621	63.191	56.076	57.796	53.699
	Difference in value	1.032	4.372	0.242	1.174	0.358	3.196	1.785	1.436	2.735
	bs standard error	0.446	4.399	1.614	0.988	1.346	3.040	1.834	2.581	1.945
	Z value	2.31 **	0.99	0.15	1.19	0.27	1.05	0.97	0.56	1.41
Organizational Leadership	MSI	60.608	59.354	60.565	62.055	57.816	67.382	58.498	60.818	56.443
	Non-MSI	58.990	58.546	60.633	59.760	57.618	60.296	57.837	57.829	54.770
	Difference in value	1.618	0.784	−0.041	2.225	0.209	7.127	0.693	2.991	1.802
	bs standard error	0.464	4.104	1.886	1.001	1.466	3.247	1.606	3.175	1.438
	Z value	3.48 ***	0.19	−0.02	2.22 **	0.14	2.2 **	0.43	0.94	1.25
Learning Satisfaction	MSI	61.264	56.909	58.741	61.698	60.942	62.217	63.086	64.217	61.112
	Non-MSI	59.902	55.583	59.678	58.024	59.711	64.493	57.727	57.416	59.400
	Difference in value	1.363	1.326	−0.965	3.723	1.231	−0.768	5.219	6.801	1.939
	bs standard error	0.530	5.784	2.170	1.223	1.446	3.322	2.239	2.918	2.404
	Z value	2.57 **	0.23	−0.44	3.05 ***	0.85	−0.23	2.33 **	2.33 **	0.81

Note: *** *p* < 0.001, ** *p* < 0.01, * *p* < 0.05. The comparison group for the PSM model and the matching variables for the logit model are the same as those in the table.

**Table 6:** Impact of MSIs on the learning and development of minority college students.

	Learning Motivation	Interpersonal Relationships	Active and Cooperative Learning	High-Impact Educational Activities	Knowledge and Skills	Innovative Thinking	Organizational Leadership	Learning Satisfaction
Ethnic Minorities	——	▼	▲	▲	▲	▲	▲	▲
Korean	——	——	——	——	——	——	——	——
Mongol	——	——	——	▲	▲	——	——	——
Hui	▲	▼	——	▲	▲	——	▲	▲
Zhuang	——	▼	——	▲	——	——	——	——
Uyghur	——	——	▲	——	▲	——	▲	——
Miao	——	——	——	▲	——	——	——	▲
Yi	——	——	——	——	——	——	——	▲
Zang	——	——	▲	▲	▲	——	——	——

Note: ▲ represents a positive influence, ▼ represents a negative influence, and — represents no significant influence.

## Data Availability

The data are available from the authors upon reasonable request.

## References

[B1-behavsci-15-00096] Adan A., Felner R. (1995). Ecological congruence and adaptation of minority youth during the transition to college. Journal of Community Psychology.

[B2-behavsci-15-00096] Atkins D. N., Fertig A. R., Wilkins V. M. (2014). Connectedness and expectations: How minority teachers can improve educational outcomes for minority students. Public Management Review.

[B3-behavsci-15-00096] Ausubel D. P. (1964). How reversible are the cognitive and motivational effects of cultural deprivation? Implications for teaching the culturally deprived child. Urban Education.

[B4-behavsci-15-00096] Boland W. C., Gasman M., Samayoa A. C., Bennett D. (2021). The effect of enrolling in minority serving institutions on earnings compared to non-minority serving institutions: A college scorecard analysis. Research in Higher Education.

[B5-behavsci-15-00096] Brown S. E., Santiago D. A., Lopez E. J. (2003). Latinos in higher education: Today and tomorrow. Change: The Magazine of Higher Learning.

[B6-behavsci-15-00096] Cabrera A., Nora A., Terenzini P., Pascarella E., Hagedorn L. (1999). Campus racial climate and the adjustment of students to college: A comparison between white students and African-American students. The Journal of Higher Education.

[B7-behavsci-15-00096] Dee T. S. (2004). Teachers, race, and student achievement in a randomized experiment. Review of Economics and Statistics.

[B8-behavsci-15-00096] DeSousa D. J., Kuh G. D. (1996). Does institutional racial composition make a difference in what black students gain from college. Journal of College Student Development.

[B9-behavsci-15-00096] Driessen G. (2015). Teacher ethnicity, student ethnicity, and student outcomes. Intercultural Education.

[B10-behavsci-15-00096] Egalite A. J., Kisida B., Winters M. A. (2015). Representation in the classroom: The effect of own-race teachers on student achievement. Economics of Education Review.

[B11-behavsci-15-00096] Ehrenberg R. G., Goldhaber D. D., Brewer D. J. (1995). Do teachers’ race, gender, and ethnicity matter? Evidence from the national educational longitudinal study of 1988. ILR Review.

[B12-behavsci-15-00096] Espinosa L. L., Turk J. M., Taylor M. (2017). Pulling back the curtain: Enrollment and outcomes at minority serving institutions.

[B13-behavsci-15-00096] Flores S., Park T. (2015). The effect of enrolling in a Minority-Serving Institution for Black and Hispanic students in Texas. Research in Higher Education.

[B14-behavsci-15-00096] Flores S., Park T., Baker D. (2017). The racial college completion gap: Evidence from Texas. Journal of Higher Education.

[B15-behavsci-15-00096] Flowers L. A., Pascarella E. T. (1999). Cognitive effects of college racial composition on African American students after 3 years of college. Journal of College Student Development.

[B16-behavsci-15-00096] Fosnacht K., Nailos J. N. (2015). Impact of the environment: How does attending a hispanic-serving institution influence the engagement of baccalaureate-seeking Latina/o students?. Journal of Hispanic Higher Education.

[B17-behavsci-15-00096] Gardner-Kitt D. (2005). Black student achievement: The influence of racial identity, ethnic identity, perception of school climate, and self-reported behavior. Ph.D. Thesis.

[B18-behavsci-15-00096] Guiffrida D. A. (2003). African American student organizations as agents of social integration. Journal of College Student Development.

[B19-behavsci-15-00096] Hanushek E. A., Kain J. F., Rivkin S. G. (2004). Why public schools lose teachers. Journal of Human Resources.

[B20-behavsci-15-00096] Harmon N. (2012). The role of minority-serving institutions in national college completion goals.

[B21-behavsci-15-00096] Hossler D., Vesper N. (1999). Going to college: How social, economic, and educational factors influence the decisions students make.

[B22-behavsci-15-00096] Hue M., Kennedy K. J. (2014). Creating culturally responsive environments: Ethnic minority teachers’ constructs of cultural diversity in Hong Kong secondary schools. Asia Pacific Journal of Education.

[B23-behavsci-15-00096] Hurtado S., Ponjuan L. (2005). Latino educational outcomes and the campus climate. Journal of Hispanic Higher Education.

[B24-behavsci-15-00096] Kim M., Conrad C. (2006). The impact of historical Black colleges and universities on the academic success of African-American students. Research in Higher Education.

[B25-behavsci-15-00096] Kuh G. D. (2008). Excerpt from high-impact educational practices: What they are, who has access to them, and why they matter. Association of American Colleges and Universities.

[B26-behavsci-15-00096] Kuh G. D., Kinzie J., Buckley J., Bridges B. K., Hayek J. C. (2006). What matters to student success: A review of the literature.

[B27-behavsci-15-00096] Kuperminc G. P., Leadbeater B. J., Emmons C., Blatt S. J. (1997). Perceived school climate and difficulties in the social adjustment of middle school students. Applied Developmental Science.

[B28-behavsci-15-00096] Luo M., Deng X., Shi J., Wei Y. (2021). The current situation and characteristics of the sense of gain among ethnic college students. Research on Ethnic Higher Education.

[B29-behavsci-15-00096] Mayhew M., Rockenbach A., Bowman N., Seifert T., Wolniak G., Pascarella E., Terenzini P. (2016). How college affects students: 21st century evidence that higher education works.

[B30-behavsci-15-00096] McDermott R. (1977). Social relations as contexts for learning in school. Harvard Educational Review.

[B31-behavsci-15-00096] Melguizo T. (2008). Quality matters: Assessing the impact of attending more selective institutions on college completion rates of minorities. Research in Higher Education.

[B32-behavsci-15-00096] Merisotis J., McCarthy K. (2005). Retention and student success at minority-serving institutions. New Directions for Institutional Research.

[B33-behavsci-15-00096] Nelson Laird T., Bridges B., Morelon-Quainoo C., Williams J., Holmes M. (2007). African American and Hispanic student engagement at minority serving and predominantly White institutions. Journal of College Student Development.

[B34-behavsci-15-00096] Oates G. L. S. C. (2003). Teacher-student racial congruence, teacher perceptions, and test performance. Social Science Quarterly.

[B35-behavsci-15-00096] Ogbu J. U. (1982). Cultural dscontinuities and schooling. Anthropology & Education Quarterly.

[B36-behavsci-15-00096] Ogbu J. U. (1987). Variability in minority school performance: A problem in search of an explanation. Anthropology & Education Quarterly.

[B37-behavsci-15-00096] Ogbu J. U. (1991). Minority coping responses and school experience. Journal of Psychohistory.

[B38-behavsci-15-00096] Park T., Flores S. (2013). Race, ethnicity, and college success: Examining the continued significance of the minority serving institution. Educational Researcher.

[B39-behavsci-15-00096] Pascarella E. T., Paulsen M. B., Perna L. W. (2019). Assessing the impact of college on students: A four-decade quest to get it approximately right. Higher Education: Handbook of Theory and Research: Volume 34.

[B40-behavsci-15-00096] Pascarella E. T., Terenzini P. T. (2005). How college affects students: A third decade of research.

[B41-behavsci-15-00096] Philips S. U. (1992). The invisible culture: Communication in classroom and community on the warm springs Indian reservation.

[B42-behavsci-15-00096] Rowley S., Moore J. (2002). Racial identity in context for the gifted African American student. Roeper Review.

[B43-behavsci-15-00096] Sherman T. M., Giles M. B., Williams-Green J. (1994). Assessment and retention of Black students in higher education. Journal of Negro Education.

[B44-behavsci-15-00096] Solorzano D., Ceja M., Yosso T. (1999). Critical race theory, racial microaggressions, and campus racial climate: The experiences of African American college students. The Journal of Negro Education.

[B45-behavsci-15-00096] Spindler G. D. (1987). Why have minority groups in North America been disadvantaged by their schools. Education and Cultural Process: Anthropological Approaches.

[B46-behavsci-15-00096] Strauss L. C., Volkwein J. F. (2002). Comparing student performance and growth in 2-and 4-year institutions. Research in Higher Education.

[B47-behavsci-15-00096] Terenzini P. T., Yaeger P. M., Bohr L., Pascarella E. T., Amaury N. (1997). African American college students’ experiences in HBCUs and PWIs and learning outcomes.

[B48-behavsci-15-00096] Thomas W. P., Collier V. P. (2002). A national study of school effectiveness for language minority students’ long-term academic achievement.

[B49-behavsci-15-00096] Tinto V. (2012). Leaving college: Rethinking the causes and cures of student attrition.

[B50-behavsci-15-00096] Upton J. N., Fleming J. (1984). Blacks in college: A comparative study of students’ success in Black and in White institutions. Contemporary Sociology.

[B51-behavsci-15-00096] Valentine C. A. (1968). Culture and poverty: Critique and counter-proposals. Current Anthropology.

[B52-behavsci-15-00096] Venezia A., Callan P. M., Finney J. E., Kirst M. W., Usdan M. D. (2005). The Governance divide: A report on a four-state study on improving college readiness and success.

[B53-behavsci-15-00096] Wang S., Zou X. (2016). Research on the influencing factors of learning engagement and learning outcomes among ethnic college students: Taking N Ethnic University as an example. Journal of Central South University for Nationalities (Humanities and Social Sciences Edition).

[B54-behavsci-15-00096] Watson L. W., Kuh G. D. (1996). The Influence of dominant race environments on student involvement, perceptions, and educational gains: A look at historically Black and predominantly White liberal arts institutions. Journal of College Student Development.

[B55-behavsci-15-00096] Wei M., Ku T. Y., Liao K. (2011). Minority stress and college persistence attitudes among African American, Asian American, and Latino students: Perception of university environment as a mediator. Cultural Diversity & Ethnic Minority Psychology.

[B56-behavsci-15-00096] Yosso T. J. (2005). Whose culture has capital? A critical race theory discussion of community cultural wealth. Race ethnicity and education.

[B57-behavsci-15-00096] Yosso T. J., Burciaga R. (2016). Reclaiming our histories, recovering community cultural wealth. Center for Critical Race Studies at UCLA Research Brief.

